# Are Older Adults Ready For Augmented Reality To Be The New FaceTime?

**DOI:** 10.1093/geroni/igaf122.3435

**Published:** 2025-12-31

**Authors:** Mahrukh Tauseef, Zalen Ingram, Rendong Zhang, Cathy Maxwell, Lisa Juckett, Judith Tate, Lorraine Mion, Nilanjan Sarkar

**Affiliations:** Vanderbilt University, Nashville, Tennessee, United States; Vanderbilt University, Nashville, Tennessee, United States; Vanderbilt University, Nashville, Tennessee, United States; University of Utah, Salt Lake City, Utah, United States; The Ohio State University, Columbus, Ohio, United States; The Ohio State University, Columbus, Ohio, United States; The Ohio State University, Columbus, Ohio, United States; Vanderbilt University, Nashville, Tennessee, United States

## Abstract

Rapid technological advances in augmented reality (AR) expand on existing video technologies (e.g., FaceTime), by offering greater social presence, realism, and immersion. Thus, AR is likely to enhance social connectedness and reduce isolation for older adults. However, older adults are often excluded during product development and/or testing. We conducted a pilot study with 6 pairs of older adults and designated family/friend to determine acceptability of AR telepresence. Each pair engaged in a shared activity through their photorealistic avatars during 8 sessions over 4 weeks. Photorealistic avatars were co-created with each participant, who provided feedback and approval on theirs and their partner’s avatars. We developed shared AR activities (checkers and home decoration) in earlier work. Data were collected at baseline, each session, and final session using surveys, semi-structured interviews, and observations. All participants completed all sessions with zero dropouts and no incidences of AR sickness. Four (25%) wanted more than one iteration to create an avatar meeting their approval. All expressed a positive attitude towards the AR experiences, e.g., “I could use this to engage with my friend in Texas” and “I like it better than video call because I can see them here”. Three requested a video of their avatars to share with family. Two expressed interest in learning how to use AR to connect with grandchildren. Challenges included a steep learning curve, limited network, and lack of realistic avatar creation tools. Nevertheless, recent improvements in network capabilities and avatar generation technology enhance AR’s likelihood as the next FaceTime.

